# Understanding the Stress Relaxation Behavior of Polymers Reinforced with Short Elastic Fibers

**DOI:** 10.3390/ma10050472

**Published:** 2017-04-28

**Authors:** Numaira Obaid, Mark T. Kortschot, Mohini Sain

**Affiliations:** 1 Department of Chemical Engineering and Applied Chemistry, Advanced Materials Group, University of Toronto, Toronto, ON M5S 3E5, Canada; numaira.obaid@mail.utoronto.ca; 2 Faculty of Forestry, Centre for Biocomposites and Biomaterial Processing, University of Toronto, Toronto, ON M5S 3B3, Canada; m.sain@utoronto.ca

**Keywords:** stress relaxation, fiber-reinforced composites, viscoelasticity, finite-element modelling

## Abstract

Although it has been experimentally shown that the addition of short-fibers slows the stress relaxation process in composites, the underlying phenomenon is complex and not well understood. Previous studies have proposed that fibers slow the relaxation process by either hindering the movement of nearby polymeric chains or by creating additional covalent bonds at the fiber-matrix interface that must be broken before bulk relaxation can occur. In this study, we propose a simplified analytical model that explicitly accounts for the influence of polymer viscoelasticity on shear stress transfer to the fibers. This model adequately explains the effect of fiber addition on the relaxation behavior without the need to postulate structural changes at the fiber-matrix interface. The model predictions were compared to those from Monte Carlo finite-element simulations, and good agreement between the two was observed.

## 1. Introduction

The interaction between the fiber and the matrix in a short-fiber composite is quite complex. Although the effect of fibers on static properties such as modulus and strength is well understood, it has been a challenge to understand the effect of fibers on the viscoelastic properties of short-fiber composites. These properties are extremely important in load bearing applications where there is the potential for creep or stress relaxation, or where the composites are exposed to any sort of dynamic loading, and hence it is important to be able to predict the influence of fiber reinforcement on the viscoelasticity. Composite viscoelasticity can also influence fatigue behavior [1], and the temperature dependence of various mechanical properties, including creep resistance [2].

Stress relaxation experiments, in which a specimen is strained to a fixed level and the slow decay of stress is monitored, present a simple method of investigating the time-dependent modulus of reinforced polymers. In practice, stress relaxation influences the residual stress and warpage of molded short-fiber composite parts, and is critical in many applications including fasteners and gaskets. During the stress relaxation of polymer composites, the modulus of the material typically decays from an initial value *E*_0_, to a final stable value *E*_∞_. The speed of this process, which has practical implications, is characterized in terms of a relaxation time constant *τ*. The time constant is usually defined as the time needed for the modulus to decrease to 1/e of the interval between *E*_0_ and *E*_∞_

In continuous fiber composites, such as laminated carbon fiber composites, the values of *E*_0_ and *E*_∞_ depend on fiber loading, but the value of *τ* should not. However, it has been widely observed that short elastic fibers (which do not themselves relax with time) alter the stress relaxation behavior of the composite, and in particular, change the value of *τ*. Early research into this phenomenon showed that short-fibers expedited the relaxation response, and many researchers proposed mechanistic explanations, in which the fibers affect the structure of the polymer matrix near the interface and hence modify its stress relaxation behavior. For example, Blackley and Pike proposed that the relaxation of composites was affected by the additional covalent bonds between the fibers and the matrix and that the rupture of these bonds during stress relaxation caused an accelerated response, changing the relaxation time constant [3]. In this and other early investigations, both the reinforcing fibers and the matrix were viscoelastic materials, and although these early studies showed that the addition of short-fibers increased the relaxation rate, almost all the more recent studies have shown the opposite effect [4,5].

Kutty and Nando investigated the effect of short Kevlar fibers on polyurethanes and found that increases in fiber content slowed the stress relaxation rate [6]. Many other studies support this observation. For example, Suhara, Kutty, and Nando also showed that increasing the loading of short polyester fibers in polyurethane resulted in slower stress relaxation [7]. Pothan et al. found that increased loading of banana fibers reduced the stress relaxation rate of polyester composites [8]. Bhattacharyya et al. also showed that increasing the content of wood fibers reduces the relaxation of polypropylene composites [9]. Saeed et al. suggested that the presence of glass fibers resulted in decreased chain mobility in high-density polyethylene [10]. Boukettaya et al. evaluated the stress relaxation behavior of polypropylene composites reinforced with date palm fibers [11]. It was observed that increasing the fiber content resulted in a decrease in the relaxation rate. Wan et al. showed that the addition of wood flour reduced the stress relaxation rate of propylene [12].

Several studies have also investigated the stress relaxation of hybrid composites containing more than one type of fiber. Sreekala et al. found that increasing the content of short oil-palm fibers in a phenol formaldehyde matrix resulted in slower stress relaxation [13]. The rate of decay could be further decreased upon hybridization with glass fibers. Stan and Fetecau investigated the stress relaxation in polytetrafluoroethylene composites [14]. Unfilled polytetrafluoroethylene (PTFE) was compared to one that was reinforced with 15% graphite particles and a hybrid containing 32% carbon and 3% graphite. It was found that unfilled PTFE had the fastest relaxation rate and that the addition of fillers slowed the relaxation process.

In summary, the literature shows that the addition of fibers to a viscoelastic polymer generally slows the relaxation process, increasing the time constant. Two main explanations have been put forward to explain this phenomenon. The first explanation is that the presence of fibers hinders molecular flow in the polymer near the interface, resulting in slower relaxation of the matrix [15]. Geethamma et al. found that short coir fibers reduced the stress relaxation rate of rubber and this was attributed to fibers constraining the polymeric chains thereby preventing relaxation [16]. Mirzaei et al. investigated the effect of adding various types of natural fibers in high-density polyethylene and drew similar conclusions [17].

An alternative explanation suggested in a number of studies centers on the potential for chemical bonding at the fiber/matrix interface. These studies propose that breaking the additional covalent bonds at the fiber-matrix interface is a prerequisite to polymer mobility and relaxation. To test this idea, a number of researchers have examined the effect of various coupling agents and their effect on stress relaxation behavior. George et al. observed that chemical modifications via coupling agents resulted in lower rates of relaxation and hypothesized that the surface treatment produces additional chemical bonds that hinder the movement of the polymer [15]. Pothan et al. also showed that the stress relaxation rate is reduced with the use of a coupling agent [7]. Boukettaya et al. proposed that from a chemical bonding perspective, the polymeric chains are initially constrained by the fiber; however, over time, the damage of the intermolecular linking causes the chains to once again become mobile [11]. Thus, it was proposed that the rate of stress relaxation in a composite was related to how quickly the bonds can be broken and, therefore, how quickly the polymer could become mobile again.

Experimental studies have confirmed that the viscoelasticity of short fiber composites is a significant and complex phenomenon. Although a number of qualitative explanations for the observed phenomenon have been proposed, as discussed previously, there have been relatively few attempts to derive a predictive model. Somashekar et al. and Safraoui et al. used conventional spring/dashpot models to characterize viscoelasticity, but although these phenomenological models can be used to characterize the behaviour of particular composites, they do not provide any guidance for optimizing material structure [18,19]. Drozdov et al. approached the viscoelasticity of composites by using an energy balance approach [20]. Several other groups including Naik et al., Brinson et al., and Fisher et al. have used finite element models incorporating viscoelastic matrices and short elastic fibers [21,22,23]. These models highlight the importance of matrix viscoelasticity, but do not obviate the need for a simple analytical model. 

The application of shear-lag models to describe composite viscoelasticity is very limited. Zhang and He examined the effect of nanofibers on the viscoelasticity of polymer-based composites; however, their work was focused on the assumption that the presence of nanofibers results in the creation of a third interfacial phase, and the shear-lag stress transfer in the resultant three-layer structure was modelled [24]. 

Recently, Smith et al. derived shear-lag stress transfer equations from first principles in a discontinuous fiber composite with a viscoelastic polymer matrix [25]. Because of the complexity of the Laplace transform arising from the derivation, it could not be inverted to produce a useable analytical model, which is a common issue [26,27]. A numerical solution was used to make predictions of stress along the length of the short fiber for only one set of parameters. Merodio used tensor analysis to derive 18 invariants associated with non-linear viscoelastic composite deformation [28]. Neither study yielded a simple, closed-form solution for stress relaxation, and hence they did not provide a way of investigating critical issues such as the effect of the fiber/matrix modulus ratio and fiber aspect ratio on the stress relaxation behavior.

The present study consists of two parts. We will first develop an analytical model by explicitly considering the stress relaxation of the matrix in both tension, and critically, in the shear stress transfer region. Through this approach, we will show that it is not necessary to infer structural changes at the interface to explain polymer composite stress relaxation. The success of this analytical model does not preclude the possibility that chemical or physical structural changes at or near the interface have an effect, but it does mean that these changes might not be important. The analytical model generated in this paper can be used to parametrically study the response of short-fiber composites with various fiber volume fractions and aspect ratios without reliance on numerical integration or finite element analysis. In the second part of this paper, we will compare the predictions from the analytical model to the results obtained from the Monte Carlo finite-element simulations. 

### 1.1. Polymer Viscoelasticity

In order to develop a simple model, the basic principles of polymer viscoelasticity and short-fiber reinforcement must be reviewed briefly. A stress relaxation test is a simple means of investigating the viscoelasticity of a polymer. To perform this test, a fixed tensile or compressive strain is applied to a sample, and the stress, which decays over time, is monitored. The decrease in stress at a constant strain corresponds to a decrease in the apparent modulus of the polymer. The modulus of a viscoelastic material during a stress relaxation test is often modeled using Equation (1) below:

(1) $$E\left(t\right)=E_{\infty }+\left(E_{0}-E_{\infty }\right)\exp\left(-\frac{t}{\tau }\right)$$

where (*E*_0_) and (*E*_∞_) are the instantaneous and long-term elastic modulus of the material respectively, *t* is time, and *τ* is the relaxation time constant. As discussed previously, the uncertainty in the literature concerns the origin of changes in the time constant commonly observed when fibers are added to a polymer.

In an isotropic solid, the shear modulus (*G*) and elastic modulus (*E*) are related by Poisson’s ratio (ν) as shown in Equation (2a). For an isotropic, viscoelastic material, at each point in time, the same relationship should hold, as shown in Equation (2b). Poisson’s ratio is usually considered to be constant in this treatment.

(2a) E=2G(1+v)

(2b) E(t)=2G(t)(1+v)

Thus, Equations (1) and (2) can be used to obtain the time-dependence of the shear modulus of a viscoelastic material, as shown in Equation (3).

(3a) $$2G\left(t\right)\left(1+v\right)=2G_{\infty }\left(1+v\right)+2\left(1+v\right)\left(G_{0}-G_{\infty }\right)\exp\left(-\frac{t}{\tau }\right)$$

(3b) $$G\left(t\right)=G_{\infty }+\left(G_{0}-G_{\infty }\right)\exp\left(-\frac{t}{\tau }\right)$$

### 1.2. Micromechanics of Short-Fiber Composites

**Figure 1:**
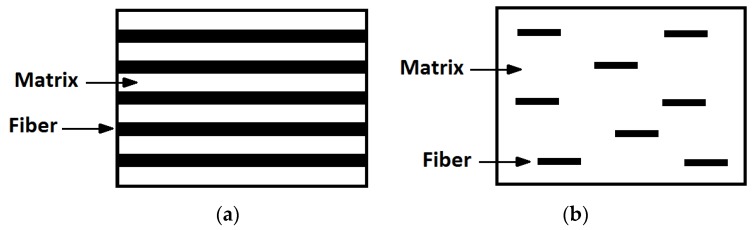
A comparison between (**a**) continuous fiber composites and (**b**) discontinuous fiber composites.

**Figure 2:**
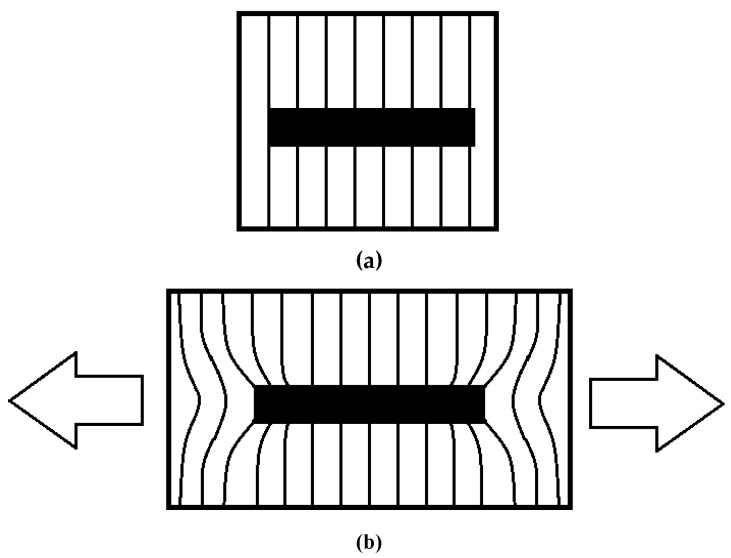
In a short-fiber composite, the matrix adjacent to the fiber is at a different stress state than the bulk matrix, resulting in a shear force along the interface. These interfacial shear stresses are responsible for stress transfer to the fibers in the composite. (**a**) Unstressed State; (**b**) Displacement under uniaxial tension.

The mechanism of fiber reinforcement in a composite depends on the aspect ratio of the fibers. Figure 1 compares the micromechanical structure of a composite reinforced with continuous and discontinuous fibers. When a continuous-fiber composite is stressed in tension, both the fiber and the matrix are equally strained. In a short-fiber composite, however, the stress required to strain the fibers is transferred through interfacial shearing (Figure 2), with the ends of the fiber being entirely unloaded. Cox developed a widely cited analytical model for the modulus of an elastic composite based on this assumption [29].

Cox’s model, which is commonly referred to as a “shear-lag” model, proposes that the effectiveness of load transfer in a short-fiber composite is related to the modulus of both the fiber and the matrix, as shown in Equations (4) and (5).

(4) $$E_{c}=V_{f}E_{f\;}\left(1-\frac{tanh\left(ns\right)}{ns}\right)+V_{m}E_{m}$$

(5) $$n={\left[\frac{4G_{m}}{E_{f}\ln\left(\frac{P_{f}}{V_{f}}\right)}\right]}^{\frac{1}{2}}$$

This model relates the elastic modulus of the composite (*E_c_*) to the elastic modulus of the fiber (*E_f_*) and matrix (*E_m_*). The contribution of each component in the composite is based on its volume fraction (*V_f_* and * V_m_*). In this equation, the contribution of the fiber is scaled by a multiplication factor (*n*) which represents the effectiveness of the load transfer to the fibers with a specified aspect ratio (*s*) and packing (*P_f_*). The load transfer depends on the ratio of the tensile modulus of the fiber to the shear modulus (*G_m_*) of the matrix, as well as the fiber aspect ratio.

Nairn identified some limitations of Cox’s shear-lag model and derived an alternate effectiveness factor [30]. However, when the ratio of the matrix to fiber modulus is sufficiently high that the shear deformation within the fiber is not significant, the predictions of Cox’s shear-lag model have been found to be reasonably accurate [31,32] and have shown good agreement with the widely used Halpin-Tsai model [33,34,35]. In this study, the matrix to fiber modulus ratio was very high, and thus Cox’s shear-lag model was deemed to be adequate for the present purposes.

### 1.3. Modelling Approach

The shear lag model makes it clear that the shear modulus of the matrix is a critical factor in determining the effectiveness of fiber reinforcement and hence the modulus of a short-fiber composite. In a viscoelastic polymer, it is well known that the effective tensile modulus decays with time in stress relaxation, and it is obvious that the effective shear modulus must also decay. It is extremely surprising therefore that virtually all previous studies of the stress relaxation of short-fiber composites have overlooked the time-dependence of the shear modulus. 

In this paper, we show that the stress-relaxation behaviour in a short-fiber composite is significantly affected by the relaxation of the shear modulus of the matrix. In addition to a decay in matrix modulus over time, relaxation of the shear modulus means that the load transfer to the fibers is decreased over time, decreasing their contribution, even when the fibers are purely elastic. However, this typically happens more slowly than the relaxation of the polymer tensile modulus, leading to a predicted increase in the effective time constant for the composite without any need to hypothesize chemical or physical structural changes at the interface.

## 2. Proposed Model

The modulus of a perfectly bonded short-fiber composite (with a fiber aspect ratio and *E_f_*/*E_m_* ratio sufficiently high that shear deformation within the fiber can be ignored) can be calculated using Equations (4) and (5).

The time-dependence of the elastic and shear moduli can be calculating using Equations (1) and (3b) assuming that the matrix is an isotropic, viscoelastic material. 

Combining Equations (1) and (4), the time-dependent elastic modulus of the composite can be written as Equation (6b).

(6a) $$E_{c}\left(t\right)=V_{f}E_{f\;}\left(1-\frac{tanh\left(ns\right)}{ns}\right)+V_{m}E_{m}\left(t\right)$$

(6b) $$E_{c}\left(t\right)=V_{f}E_{f\;}\left(1-\frac{tanh\left(ns\right)}{ns}\right)+V_{m}\left[E_{inf}+\left(E_{0}-E_{\infty }\right)\exp\left(-\frac{t}{\tau }\right)\right]$$

However, the stress-transfer to the fiber is also time-dependent due to the time-dependence of the matrix shear modulus. Thus, the stress-transfer coefficient in Equation (5) can be combined with Equation (3b) as shown in Equation (7).

(7a) $$n={\left[\frac{4}{E_{f}\ln\left(\frac{P_{f}}{V_{f}}\right)}\right]}^{\frac{1}{2}}{\left[G_{m}\left(t\right)\right]}^{\frac{1}{2}}$$

(7b) $$n={\left[\frac{4}{E_{f}\ln\left(\frac{P_{f}}{V_{f}}\right)}\right]}^{\frac{1}{2}}{\left[G_{\infty }+\left(G_{0}-G_{\infty }\right)\exp\left(-\frac{t}{\tau }\right)\right]}^{\frac{1}{2}}$$

The time-dependent elastic modulus of a short-fiber composite is therefore fully characterized by Equation (8).

(8a) $$E_{c}\left(t\right)=V_{f}E_{f\;}\left(1-\frac{tanh\left(n\left(t\right)s\right)}{n\left(t\right)s}\right)+V_{m}\left[E_{inf}+\left(E_{0}-E_{\infty }\right)\exp\left(-\frac{t}{\tau }\right)\right]$$

(8b) $$n\left(t\right)={\left[\frac{4}{E_{f}\ln\left(\frac{P_{f}}{V_{f}}\right)}\right]}^{\frac{1}{2}}{\left[G_{\infty }+\left(G_{0}-G_{\infty }\right)\exp\left(-\frac{t}{\tau }\right)\right]}^{\frac{1}{2}}$$

Equation (8) predicts the composite modulus decay, *E_c_*(*t*), as a function of the time dependent tensile and shear moduli of the matrix. 

## 3. Parametric Study

### 3.1. Properties of the Matrix and Fiber

Equation (8) was used to make modulus predictions for a glass-fiber reinforced polyurethane composite. The glass fiber was assigned an elastic modulus of 80 GPa, typical of that reported in the literature. For simplicity, the fibers were assumed to have a packing factor of 1 (hexagonal packing). The Poisson’s ratio of the matrix was assumed to be 0.3. The fiber modulus was two orders of magnitude above that of the PU matrix, and hence the Cox model was sufficient for the shear lag computations needed in this study.

### 3.2. Effect of Fiber Content

**Figure 3:**
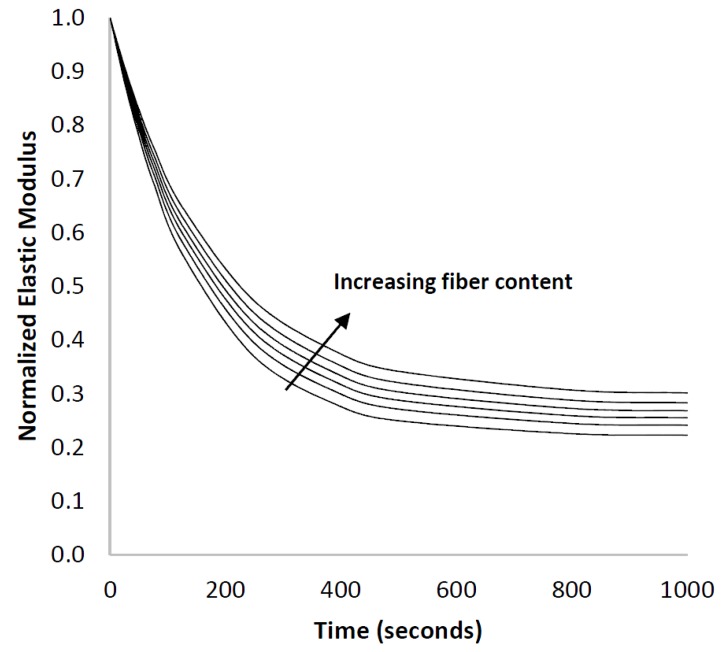
The normalized elastic modulus of the polyurethane-glass composites under stress-relaxation. The fiber content ranges from 0% to 50%. The initial elastic modulus depends on the fiber content, but the data here have been normalized by the modulus of the unreinforced polymer so that the stress relaxation is highlighted.

**Figure 4:**
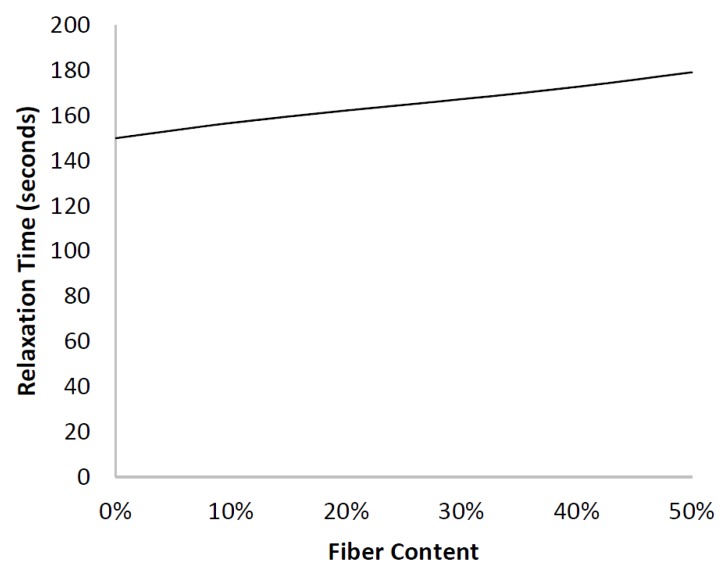
Higher fiber contents resulted in an increase in the relaxation time constant indicating that the rate of relaxation had slowed. This showed that increasing the fiber fraction slowed the relaxation of the composite.

The modulus of the composite at various fiber fractions was calculated using Equation (8) and is shown in Figure 3. In order to further examine the rate of relaxation, the predicted values were fitted to Equation (1) to determine the relaxation rate constant (*τ*) and the fractional deterioration in the modulus of the composite (*E*_0_ − *E*_∞_)/*E*_0_, as shown in Figure 4. For this part of the study, the matrix was assumed to have an instantaneous modulus of 450 MPa, a long-term elastic modulus of 100 MPa, and a relaxation time constant of 150 s. The fiber was assumed to have an aspect ratio of 10.

Figure 3 shows that the model predicts a change in the shape of the stress-relaxation curve upon the addition of purely elastic fibers to a viscoelastic matrix, affecting the rate at which the modulus deteriorates. If the elastic fibers did not introduce any additional viscoelastic effects, their presence would result in equal reinforcement at each instant in time causing no change in the relaxation time constant (*τ*) [36]. However, the model shows that increasing the fiber content resulted in a longer relaxation time constant, indicating that the presence of fibers slowed the rate of relaxation. It is precisely this change that elicited the various mechanistic explanations previously reported in the literature, but Figure 4 demonstrates that the change is a simple consequence of time dependent shear stress transfer to short-fibers. Of course, these results do not prove that there are no structural changes at the fiber interface, only that they are not necessary to produce the observed behaviour.

### 3.3. Effect of Fiber Aspect Ratio

**Figure 5:**
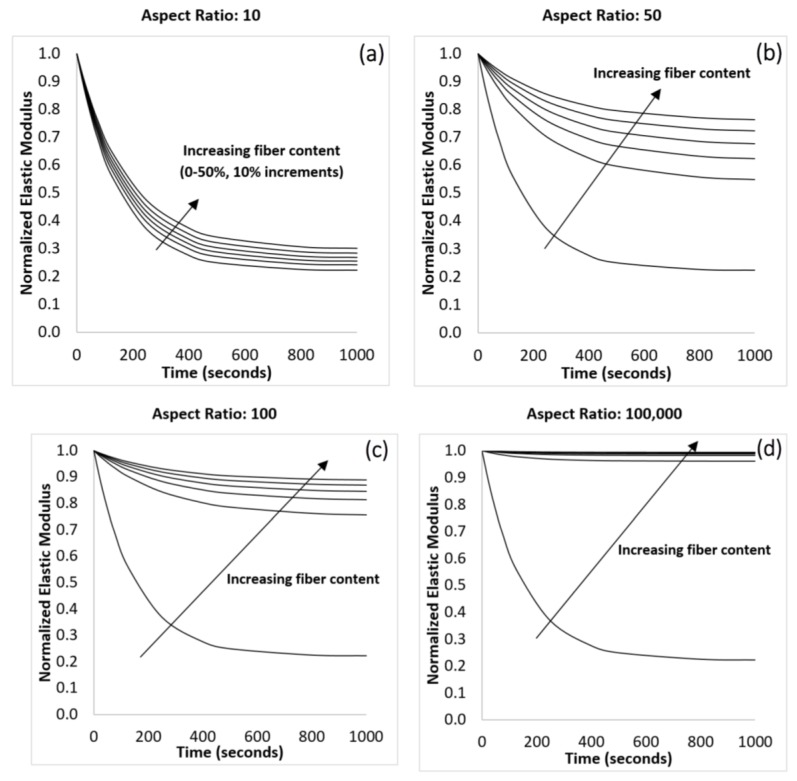
This graph depicts the change in normalized elastic modulus with fiber content at various fiber aspect ratios including (**a**) aspect ratio of 10; (**b**) aspect ratio of 50; (**c**) aspect ratio of 100 and (**d**) aspect ratio of 100,000. It can be observed that as the fiber aspect ratio is increased, the long-term modulus increases because the longer fibers are more efficient reinforcements.

We also investigated the effect of the fiber aspect ratio on stress relaxation of the polyurethane-glass fiber composites. For this part of the study, the matrix was assumed to have an instantaneous modulus of 450 MPa, a long-term elastic modulus of 100 MPa, and a relaxation time constant of 150 s. The normalized relaxation modulus with various fiber contents and fiber aspect ratios is shown in Figure 5.

It was observed that increasing the fiber aspect ratio resulted in an increase in the long-term relaxation modulus of the composite (in comparison to the modulus of the unreinforced polymer, all the data is normalized). This indicated that higher aspect ratio fibers more effectively reduce the stress relaxation. As the aspect ratio of the fibers increases, the behavior of the composite approaches that of a continuous fiber composite.

**Figure 6:**
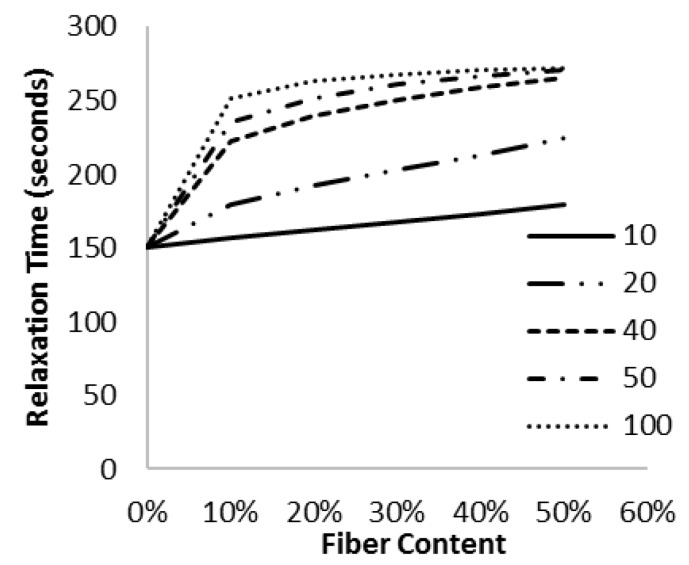
As the aspect ratio of the fiber increases, more load is transferred from the matrix to the fiber. This increased shear force on the fiber results in a higher relaxation time.

**Figure 7:**
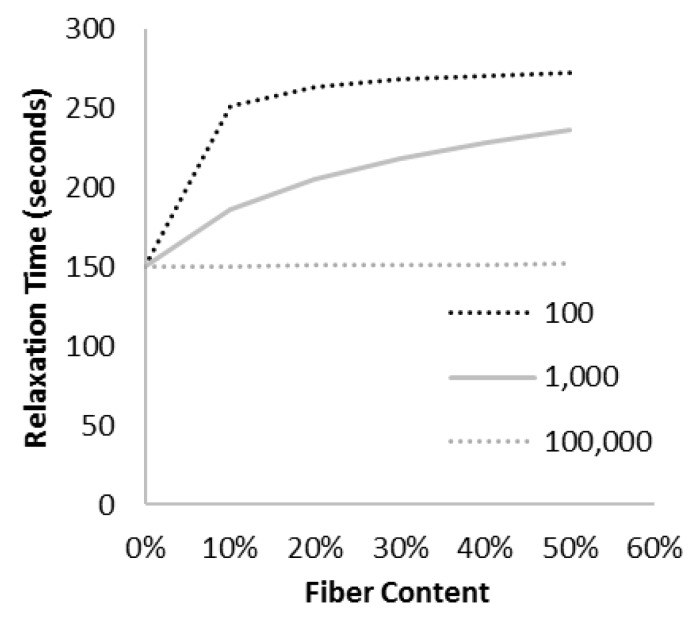
As the aspect ratio is continually increased, a larger fraction of the fiber is under tensile loading and the influence of the shear loading zone decreases. At very high aspect ratios, the composite begins to approach the properties of a continuous fiber composite, with no change in the relaxation time.

The effect of the fiber aspect ratio on the relaxation time constant was extracted from the data in Figure 5 and is shown in Figure 6 and Figure 7.

It appears that changing the fiber aspect ratio results in two regimes: for a fiber aspect ratio below a critical value, increasing the aspect ratio increases the relaxation time. However, the model also predicts that when the aspect ratio is above a critical value, the opposite effect will occur. For the modulus ratio (*E_f_/G_m_*) used here, the transition occurs at an aspect ratio of ~100. The critical aspect ratio for viscoelasticity may now be defined as the aspect ratio corresponding to the maximum relaxation time. Expressed another way, this is the aspect ratio for which the shear stress transfer into the fibers is the most critical to stress relaxation of the composite. This result is surprising, but is a simple outcome of shear lag analysis incorporating a time dependent shear modulus.

**Figure 8:**
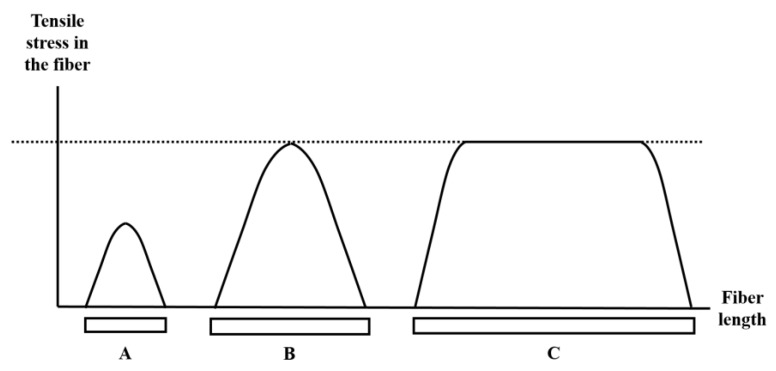
The tensile stresses in the fiber are dependent on its aspect ratio. The aspect ratio (**B**) is the value at which the maximum stress transfer begins to occur in the fiber. If the aspect ratio of the fibers is too low (**A**), there is inadequate stress transfer between the fiber and matrix. If the aspect ratio is too high (**C**), the properties of the composite approach that of a long-fiber composite.

For very low aspect fibers, there is little stress transfer to the fiber. Consequently, the matrix dominates the behaviour, and the addition of fibers has little effect on the composite viscoelasticity. The numerical value of a “low” aspect ratio depends entirely on the modulus ratio *E_f_/G_m_*, but simply means that the fiber is not effective because it is not long enough to be strained effectively (see Figure 8).

As the fiber aspect ratio increases, the fiber takes a larger fraction of the composite load, and the time-dependent shear stress transfer between the matrix and fiber becomes important, so there is a significant change in the time constant as we add more fibers to the mix.

As the aspect ratio increases further, the composite begins to resemble a long fiber composite, and the shear stress transfer is no longer important since it is confined to end sections that are a trivial fraction of the overall fiber length [29]. Once the shear modulus of the matrix is not important, the model developed here is not needed, and as expected, the relaxation time constant for a continuous fiber composite (aspect ratio of 100,000 in Figure 7) is not affected by fiber content.

None of this behaviour has anything to do with chemical bonding or chain mobility hindrance at the fiber matrix interface; these phenomena are not affected by the fiber aspect ratio, and of course, are not part of the model.

## 4. Monte Carlo Finite-Element Analysis

### 4.1. Modelling Approach

To confirm the analytical results, finite element analysis using multiple trials with randomly deposited short fibers was conducted in Abaqus CAE. For this work, the matrix was defined as a viscoelastic material with an instantaneous elastic modulus of 1000 MPa, a long-term modulus of 500 MPa, a relaxation-time constant of 100 s, and a Poisson’s ratio of 0.5. The fibers were defined as E-glass fibers having an elastic modulus of 80 GPa and a Poisson’s ratio of 0.2; these values were obtained from the literature [37]. The fibers selected for this study had a diameter of 16 microns and a length of 260 microns. 

The model consisted of a three-component system including a matrix, fibers, and a rigid body used to apply fixed displacement to the upper surface of the specimens, corresponding to a stress relaxation experiment. The matrix was defined as a 3D deformable object while the fibers were defined as beam elements with circular cross-sections for computational efficiency. An embedded constraint was applied between the matrix and fibers representing a perfect bond. The total number of fibers dispersed in the system was adjusted to represent various fiber volume fractions. The matrix mesh consisted of 2211 3D standard quadratic (C3D20R) elements while the fiber mesh consisted of 40 standard quadratic beam elements (B32) per fiber. 

For each finite-element run, the positions of the fiber centres were randomly generated using a Python script. All fibers were aligned in the load direction. Five replicate simulations with differing but random fiber locations were conducted for each volume fraction.

The finite-element analysis stress-relaxation test consisted of two analysis steps: the instantaneous application of strain, followed by 400 s of stress decay monitoring, while the mesh was held at fixed deformation. The modulus of the composite was determined in the conventional way using the cross sectional area, and the applied force and displacement of the rigid body. This approach was validated by comparing the input modulus to the calculated modulus from the simulation outputs for an isotropic one-phase system. The data was obtained at 10-s intervals with a minimum increment time step of 0.004 s.

### 4.2. FEA Results

**Figure 9:**
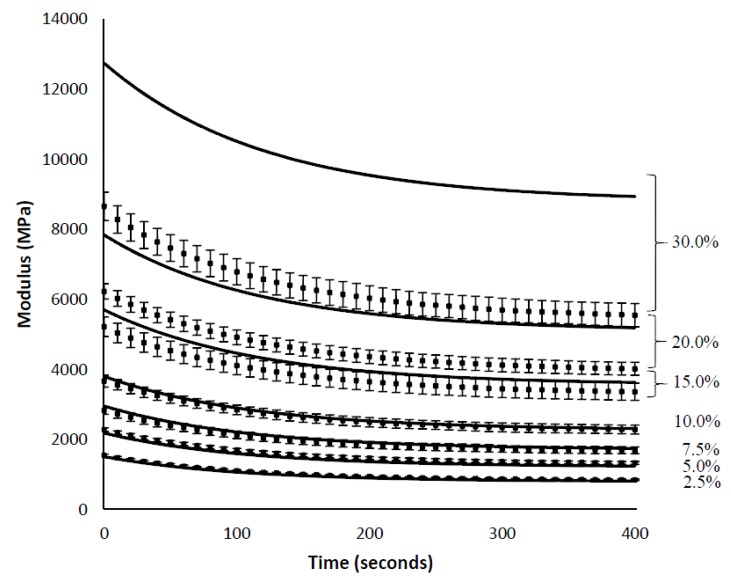
A comparison of the overall stress relaxation profile of short-fiber composites shows excellent agreement between the predictions of the analytical model (-) and the results obtained from the finite-element simulations (▪). The error bars represent the standard deviation resulting from five runs of the FEA model material with differing random fiber placements.

The analytical model was re-evaluated using a set of material properties identical to that used in the finite-element simulations (see Section 4.1) and a comparison of the results is shown in Figure 9. Excellent agreement is observed between the finite-element simulations and the predictions made using the analytical model for fiber contents up to 15% (by volume). This agreement supports the validity of the analytical model, and a key finding is that both the FEA and the analytical model yielded changes in the relaxation time constant with fiber loading.

In Figure 9, it is apparent that the analytical model overpredicts the modulus at all times for fiber loadings greater than 15%. The Cox shear lag model, upon which the current model is based, assumes that each fiber is sitting in an isolated pocket of resin, and that everywhere within the perimeter of the pocket is experiencing the remote strain. At higher volume fractions, the situation is clearly more complex than this, as fibers approach and even touch each other, and the model is not expected to be accurate. In fact, studies that have compared the elastic modulus for composites have shown that the shear-lag model often overpredicts the actual modulus [38]. 

**Figure 10:**
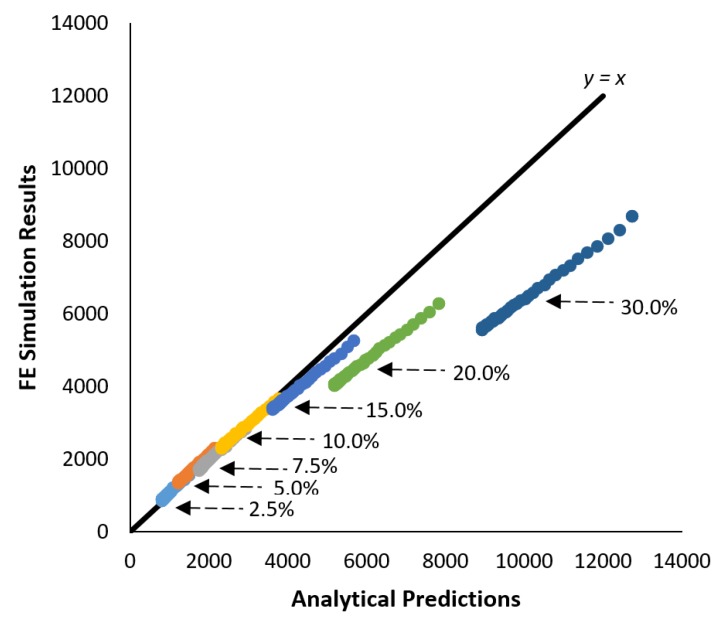
Comparison of the analytical model predictions to the finite-element simulation results shows good agreement between the two at low volume fraction; however, at volume fractions equal to 20% and greater, the finite-element results deviate from the predictions of the analytical model.

The fit was also examined by comparing the analytical model predictions to the finite element simulation results at the each point in time. For a good fit between the two, the two values should be almost equal, resulting in a slope close to one. Thus, closer proximity to a y = x line can represent a better fit between the two approaches (see Figure 10).

Figure 10 shows a strong agreement between the analytical model and the finite-element simulations at low fiber volume fractions as observed by their proximity to the y = x line. Since it has been determined that the shear-lag model is not applicable for fiber volume fractions greater than 20%, composites with fiber volume fractions above this threshold have not been used in further analysis.

**Figure 11:**
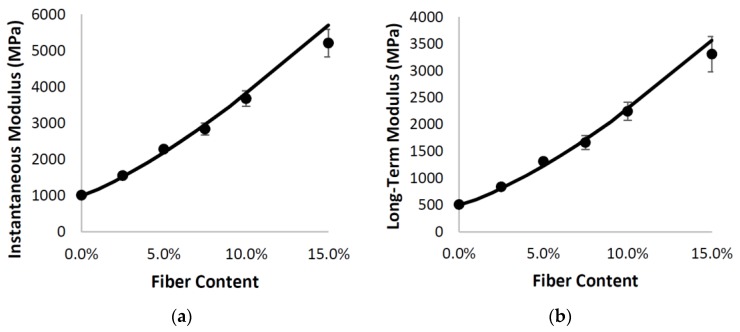
Good agreement is obtained between the instantaneous (**a**) and long-term (**b**) modulus values obtained from the analytical model (-) and the finite-element simulations (●).

The results from both the analytical model and finite-element results were fit to a simple Prony Series, and three key parameters were obtained: the instantaneous modulus, the long-term modulus, and the stress relaxation constant. The addition of elastic fibers results in an increase in both moduli as expected and there was excellent agreement between the analytical and finite element models (see Figure 11). 

**Figure 12:**
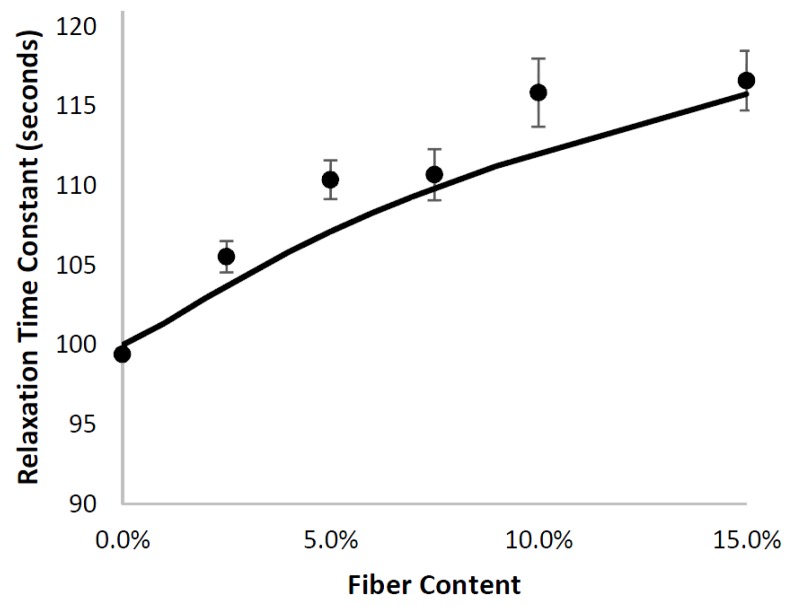
Good agreement is obtained between the relaxation time constant obtained from the analytical model (-) and the finite-element simulations (●).

In Figure 12, the clear dependency of the relaxation rate constant on fiber content is illustrated. Both the FEA and the analytical model predicted this trend. It is important to note that the analytical model indicated that the change in relaxation rate constant stemmed from the time-dependent shear modulus of the matrix, which resulted in time-dependent shear stress transfer to the fibers, causing the stress within the fiber to be time-dependent, and thus, having an indirect effect on the stress relaxation of a short-fiber composite. 

## 5. Conclusions

Although it has been experimentally shown that the presence of short-fibers slows the relaxation process in composites, the underlying phenomenon is complex and was not well understood. Previous studies have postulated either microstructural or chemical interactions between the fiber and matrix on a molecular scale in order to explain the observed changes in relaxation, but in this study, we have shown that the effect of fibers on the stress relaxation behaviour of a composite can be explained by simply considering the fundamentals of shear stress-transfer at the fiber-matrix interface in short-fiber composites. The fiber-matrix interface is simply considered to be an infinitely thin, perfectly bonded zone.

This study shows that the stress relaxation of a composite is influenced by two phenomena: firstly, the elastic modulus of the matrix is time-dependent, and secondly, the shear modulus of the matrix is also time-dependent and causes a time-dependent stress-transfer between the fiber and the matrix. As the fiber content increases, the relative importance of the shear stress transfer zone increases, causing an increase in the time constant for relaxation. This effect is largest for intermediate aspect ratios where the fibers are long enough to carry a significant fraction of the load, but short enough to be affected by the shear stress transfer from the matrix over a significant portion of their length.

The concept of a critical fiber length (or aspect ratio) is widely used with respect to the strength, modulus, and toughness of short fiber composites. We have identified a critical fiber aspect ratio for viscoelasticity as the aspect ratio for which the shear lag stress transfer zone is most influential in determining the overall load carrying ability of the composite, and hence most critical in determining the effect of fiber loading on the time constant for stress relaxation.

In summary, an explicit accounting of the relaxation of shear modulus and the effect of this on the reinforcement efficiency factor can adequately explain the effect of short fibers on stress relaxation in polymer composites without any inference of structural changes at the interface. Since viscoelastic behaviour of short fiber composites is extremely important in many applications, this model should find wide applications.
